# Chromosome Instability in the Neurodegenerating Brain

**DOI:** 10.3389/fgene.2019.00892

**Published:** 2019-09-20

**Authors:** Yuri B. Yurov, Svetlana G. Vorsanova, Ivan Y. Iourov

**Affiliations:** ^1^Yurov’s Laboratory of Molecular Genetics and Cytogenomics of the Brain, Mental Health Research Center, Moscow, Russia; ^2^Laboratory of Molecular Cytogenetics of Neuropsychiatric Diseases, Veltischev Research and Clinical Institute for Pediatrics of the Pirogov Russian National Research Medical University, Moscow, Russia

**Keywords:** brain, chromosome insatiability, neurodegeneration, pathways, aneuploidy, genome stability, somatic mosaicism

Chromosome instability (CIN) is a hallmark of cancer (Heng, 2015; Rangel et al., 2017; Machiela, 2019; Simonetti et al., 2019). Additionally, a number of neurodegenerative diseases (NDD) demonstrate CIN, which mediates neuronal cell loss and appears to be a key element of the pathogenic cascade (Iourov et al., 2009a; Iourov et al., 2009b; Arendt et al., 2010; Jeppesen et al., 2011; Driver, 2012; Bajic et al., 2015; Leija-Salazar et al., 2018; Nudelman et al., 2019). Moreover, CIN is repeatedly associated with aging and aging-related deterioration of the brain (Yurov et al., 2010; Kennedy et al., 2012; Andriani et al., 2017; Vijg et al., 2017; Zhang and Vijg, 2018). Despite numerous studies dedicated to CIN in NDD, there is still no clear understanding of differences between “cancerous” and “neurodegenerative” CINs. Here, we propose a theoretical model, which seems to highlight the differences between these CIN types.

Oncogenic parallels have long been observed in NDD. More specifically, CIN manifesting as aneuploidy (gains or losses of whole chromosomes) has been systematically identified in the brain of individuals with NDD. The Alzheimer’s disease brain has been found to demonstrate high rates of spontaneous aneuploidy (Iourov et al., 2009b; Iourov et al., 2011; Yurov et al., 2014; Bajic et al., 2015; Arendt et al., 2017; Yurov et al., 2018). Furthermore, Alzheimer’s disease genes are involved in molecular pathways, alterations to which result in chromosome mis-segregation and aneuploidy (Granic et al., 2010). Similarly, CIN syndromes and/or mutations in genes involved in cell cycle/mitotic checkpoint pathways exhibit brain-specific CIN associated with neurodegeneration. Thus, CIN has been demonstrated to underlie neurodegenerative processes (Iourov et al., 2009a; Caneus et al., 2018; Leija-Salazar et al., 2018). Additionally, submicroscopic CIN producing structural rearrangements of the *APP* gene (21q21.3) has been shown to be involved in neurodegenerative pathways to Alzheimer’s disease (Bushman et al., 2015; Lee et al., 2018). It is important to note that numerical CIN (aneuploidy) is shown to be implicated in the neurodegeneration pathway inasmuch as the neurons affected by CIN/aneuploidy are susceptible to selective cell death (Arendt et al., 2010; Fricker et al., 2018; Iourov et al., 2019). Finally, DNA repair deficiency (Jeppesen et al., 2011) and DNA replication stress (Yurov et al., 2011) have been identified as possible mechanisms for neurodegeneration.

Another body of evidence for the contribution of CIN to neurodegeneration is provided by brain aging studies. Actually, CIN and related phenomena (aneuploidization, somatic mutagenesis, etc.) are considered to be elements of a global pathogenic cascade resulting in aging phenotypes (Kennedy et al., 2012; Vijg, 2014; Andriani et al., 2017). Progressive accumulation of somatic chromosomal mutations (aneuploidy) causing numerical CIN is suggested to be implicated in cellular senescence and tissue aging (Yurov et al., 2010; Zhang and Vijg, 2018; Iourov et al., 2019). For instance, rates of X chromosome aneuploidy increase with age in the Alzheimer’s disease brain (Yurov et al., 2014). It is to note that X chromosome aneuploidy (loss/monosomy) is a cytogenetic biomarker of human aging (Vijg, 2014; Zhang and Vijg, 2018; Iourov et al., 2019). Genome instability at the chromosomal level (numerical and structural CINs) has been determined as a conserved mechanism for aging, as a whole, and, more particularly, for aging of the brain, a post-mitotic tissue with an extremely limited potential of cell renewal (Yurov et al., 2010; Andriani et al., 2017; Vijg et al., 2017). It appears that aging-related CIN leads to aging-related deterioration of the brain producing phenotypes similar to NDD (Andriani et al., 2017; Zhang and Vijg, 2018). Functionally, CIN is supposed to be an underlying cause of cellular (neuronal) senescence (Yurov et al., 2010; Arendt et al., 2017; Zhang and Vijg, 2018; Iourov et al., 2019). The latter has been recently demonstrated to represent a mechanism for both brain aging and NDD (Baker and Petersen, 2018). Therefore, one may conclude that the pathogenic pathways are likely to be shared by brain aging, neurodegeneration, and cancer.

NDD (e.g., Alzheimer’s disease) have been consistently shown to share biological hallmarks with cancer, which are, but not limited to, alterations to genome stability maintenance pathways (mitotic checkpoint, cell-cycle regulation, DNA replication/repair, programmed cell death, etc.) and CIN/genome instability (for review, see Driver, 2012, Arendt et al., 2017, Nudelman et al., 2019). More precisely, numerical CIN (aneuploidy) leading to chromosomal mosaicism is a mechanism for a variety of brain diseases including NDD. Somatic mosaicism and increased rates of aneuploidy and structural CIN have been identified in the neurodegenerating brain (Alzheimer’s disease and ataxia telangiectasia), schizophrenia brain, and individuals with intellectual disability and autism spectrum disorders. Mutations of specific genes implicated in genome stability maintenance pathways have been associated with NDD (Iourov et al., 2009a; Iourov et al., 2009b; Arendt et al., 2010; Iourov et al., 2011; Jeppesen et al., 2011; Yurov et al., 2014; Bajic et al., 2015; Caneus et al., 2018; Rohrback et al., 2018; Yurov et al., 2018; Iourov et al., 2019). Aneuploidy is a common feature of cancer cell populations and is likely to influence cancer behavior (for review, see Simonetti et al., 2019). Moreover, chromosomal mosaicism is a susceptibility factor for cancer (Schick et al., 2013; Vijg, 2014; Machiela, 2019). Genetic alterations to the genome stability maintenance pathways produced by copy number and sequence variations of the implicated genes are observed both in cancer and in the neurodegenerating brain (Granic et al., 2010; Bushman et al., 2015; Heng, 2015; Caneus et al., 2018; Lee et al., 2018). As noted before, a possible mechanism of neurodegeneration is DNA repair deficiency (Jeppesen et al., 2011). The later commonly leads to CIN and karyotypic chaos in a wide spectrum of cancers (Driver, 2012; Heng, 2015; Rangel et al., 2017). DNA replication stress seems to lie at the origins of CIN in the neurodegenerating brain of individuals with Alzheimer’s disease (Yurov et al., 2011). Likewise, this phenomenon negatively impacts chromosome segregation producing CIN during tumorigenesis (Zhang et al., 2019). Finally, cellular senescence is able to contribute both to neurodegeneration (brain aging deterioration) and to cancer (Yurov et al., 2010; Vijg, 2014; Baker and Petersen, 2018; Machiela, 2019). It appears that either neurodegeneration or cancer is more likely to result from complex genetic-environmental interactions, in which CIN plays a key role in the pathogenic cascade (Iourov et al., 2013; Heng, 2015). However, taking into account diverse consequences of “neurodegenerative” and “cancerous” CINs, there should be a number of differences between these types of chromosome/genome instability. For instance, the lack of convincing evidence for comorbidities such as NDD and brain cancers suggests that brain cells affected by CIN may have at least two alternative fates: (i) to become malignant (i.e., cancerization) and (ii) to be cleared by cell death (i.e., neurodegeneration). Therefore, there should be a striking difference in molecular pathways to cancer and NDD.

Since somatic mosaicism and CIN in the brain are more likely to have developmental origins (Yurov et al., 2007; Rohrback et al., 2018; Yurov et al., 2018; Iourov et al., 2019), alterations to programmed cell death may be an explanation of the presence of cells with abnormal chromosome complements (genomes) in the diseased brain (Arendt et al., 2010; Yurov et al., 2010; Fricker et al., 2018; Iourov et al., 2019). More precisely, abnormal neural cells generated during the development are not cleared throughout gestation and antenatal period. As a result, CIN-affected (abnormal) cellular populations alter brain functioning after birth (for more details, see Yurov et al., 2007; Yurov et al., 2010; Rohrback et al., 2018; Iourov et al., 2019). Thus, programmed cell death acts differently in the neurodegenerating brain and in cancer. The former demonstrates excessive neuronal cell loss probably mediated by CIN, whereas the latter is characterized by astonishing tolerance of cell populations to programed cell death (Heng, 2015; Fricker et al., 2018; Iourov et al., 2019). Therefore, cancer cells are likely to be affected by abnormal cell-death checkpoint in contrast to neuronal cells affected by “neurodegenerative CIN,” in which the checkpoint probably acts to an abnormal environmental trigger. Interestingly, CIN/aneuploidy is usually chromosome-specific in the diseased brain. In the Alzheimer’s disease brain, CIN commonly involves chromosome 21, whereas the selectively degenerating cerebellum of ataxia-telangiectasia individuals exhibits CIN commonly involving chromosome 14 (Iourov et al., 2009a; Iourov et al., 2009b; Arendt et al., 2010; Granic et al., 2010). This is generally not the case for the overwhelming majority of cancer cells expressing genetic defect specific for a cancer/ tumor type, karyotypic chaos, or numerical and structural CINs (Heng, 2015). The natural selection pressure against cells affected by non-specific CIN types and observations on patterns of CIN in the neurodegenerating brain suggest that neuronal cell populations affected by neurodegeneration possess primary genetic defects without progressive clonal evolution (Iourov et al., 2009a; Arendt et al., 2010; Yurov et al., 2011; Iourov et al., 2013; Arendt et al., 2017; Leija-Salazar et al., 2018). The latter, however, is shown to be an underlying cause of cancer (Driver, 2012; Heng, 2015; Rangel et al., 2017; Simonetti et al., 2019). Taking into consideration the aforementioned differences between cancer and NDD, we have proposed a theoretical model for CIN to mediate either cancer or neurodegeneration. Thus, “cancerous CIN” is likely to result from genetic-environment interactions and genetic defects, which render cells with unstable genomes tolerant to clearance (i.e., programmed cell death) and advantageous for proliferation over other cells. The malignancy is then achieved by clonal evolution. Alternatively, CIN and aneuploidy may possess a detrimental effect on cell growth under the normal growth conditions. In this case, cancerization is achieved through an adaptation of a subclone of cells to aneuploidy and CIN, which further evolves to a cell population with a fitness advantage (Vijg, 2014; Heng, 2015; Zhang et al., 2019). As a result, cells tolerating CIN without the loss form a stable cell population causing cancer invasion and metastasis (Loeb, 2010).

In contrast to cancer, neurodegeneration is likely to start because of the interaction between environmental trigger and CIN/genetic defects persisting in an appreciable proportion of brain cells. The interactions may launch a kind of “neuroprotective program” for clearance of CIN-affected cells. It appears that such “neuroprotective program” exists in the developing mammalian brain, which loses the majority of cells affected by CIN throughout gestation. It has been hypothesized that CIN/aneuploidy serves as an initiator of cell death (i.e., mitotic catastrophe) under natural selection in the developing brain (Yurov et al., 2007; Yurov et al., 2010; Rohrback et al., 2018; Iourov et al., 2019). Since CIN affects the critical number of neuronal cells (Iourov et al., 2009a), progressive loss of these cells would produce brain dysfunction leading to NDD phenotypes. **Figure 1** schematically shows our model for CIN contribution to cancer and neurodegeneration according to observations on CIN in the neurodegenerating brain in cancers (Iourov et al., 2009a; Iourov et al., 2009b; Arendt et al., 2010; Granic et al., 2010; Iourov et al., 2011; Jeppesen et al., 2011; Yurov et al., 2011; Driver, 2012; Kennedy et al., 2012; Vijg, 2014; Yurov et al., 2014; Bajic et al., 2015; Heng, 2015; Arendt et al., 2017; Rangel et al., 2017; Caneus et al., 2018; Leija-Salazar et al., 2018; Yurov et al., 2018; Machiela, 2019; Simonetti et al., 2019).

**Figure 1:**
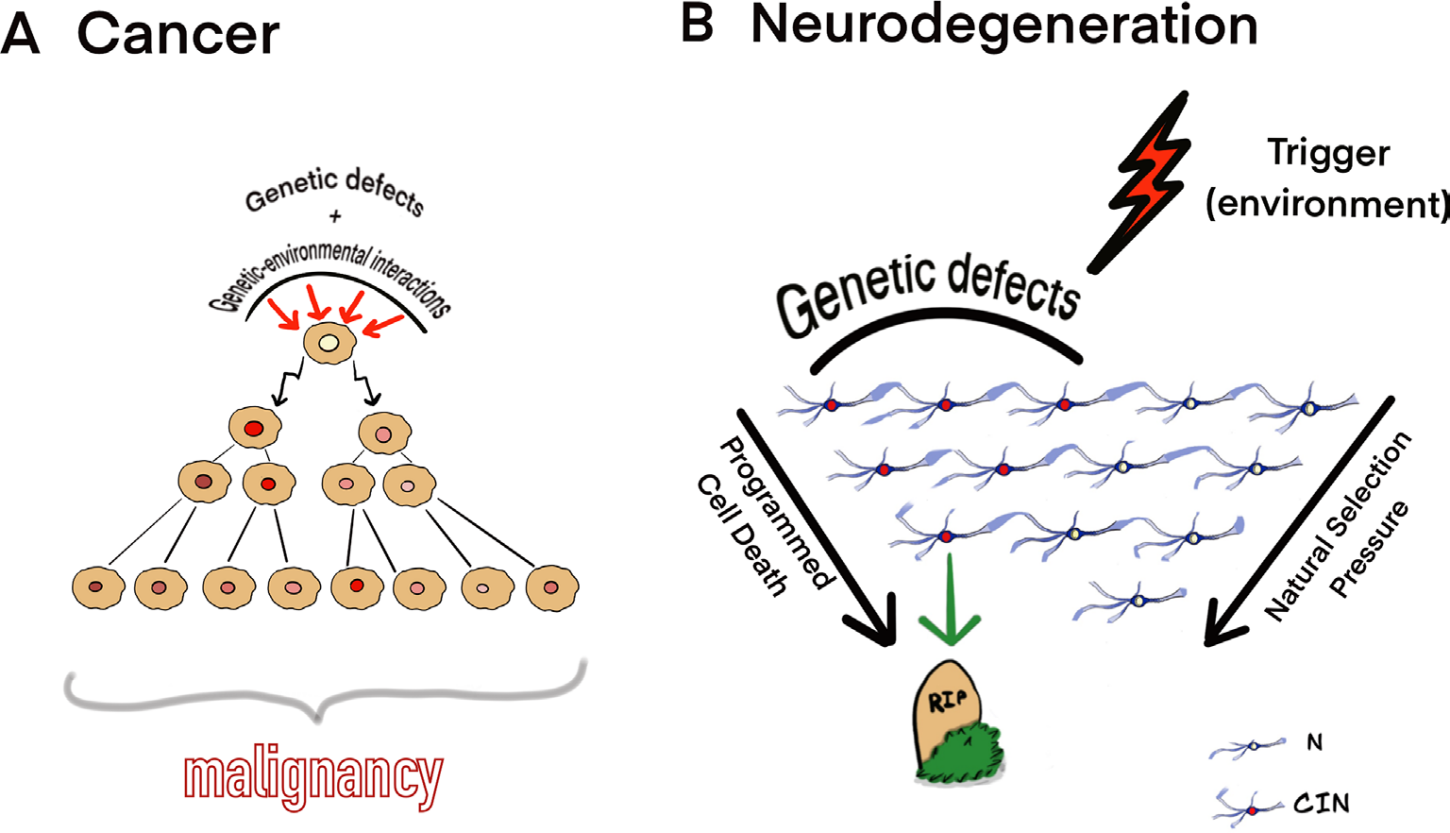
Theoretical model for CIN mediating **(A)** cancer and **(B)** neurodegeneration. **(A)** Genetic defects and genetic-environmental interactions may cause chromosomal/genomic changes, which produce CIN; alternatively, cell populations may adapt to aneuploidy and CIN evolving to a cell population with a fitness advantage. Cells affected by CIN and tolerating deteriorating effects of CIN on cellular homeostasis are able to evolve clonally to produce malignancy. **(B)** CIN/somatic mosaicism affecting a significant proportion of cells interacting with environmental triggers may result into progressive neuronal cell loss (neurodegeneration) under natural selection pressure and through the programmed cell death (N, normal neurons; CIN, neuronal cell affected by CIN). The model is based on the observations of CIN in the neurodegenerating brain and cancers (Iourov et al., 2009a; Iourov et al., 2009b; Arendt et al., 2010; Granic et al., 2010; Iourov et al., 2011; Jeppesen et al., 2011; Yurov et al., 2011; Driver, 2012; Kennedy et al., 2012; Vijg, 2014; Yurov et al., 2014; Bajic et al., 2015; Heng, 2015; Arendt et al., 2017; Rangel et al., 2017; Caneus et al., 2018; Leija-Salazar et al., 2018; Yurov et al., 2018; Machiela, 2019; Simonetti et al., 2019).

Understating the role of CIN in the neurodegeneration pathway is important for successful therapeutic interventions in NDD. Certainly, there is a need for further studies dedicated to analysis of the applicability of the “neurodegenerative CIN” model to describe molecular and cellular mechanisms for neurodegeneration. If the model is applicable, new opportunities for NDD prevention and treatments through the external control of CIN will be available.

## Author Contributions

All authors conceived the idea and made theoretical contributions to the manuscript. II wrote the manuscript.

## Funding

Authors are partially supported by RFBR and CITMA according to the research project No. 18-515-34005. Prof. SG Vorsanova is supported by the Government Assignment of the Russian Ministry of Health, Assignment no. AAAA-A18-118051590122-7. Prof. IY Iourov is supported by the Government Assignment of the Russian Ministry of Science and Higher Education, Assignment no. AAAA-A19-119040490101-6.

## Conflict of Interest Statement

The authors declare that the research was conducted in the absence of any commercial or financial relationships that could be construed as a potential conflict of interest.
